# Impact of R0 resection for synchronous peritoneal metastasis from colorectal cancer: A propensity score‐matched analysis of a multi‐institutional database

**DOI:** 10.1002/ags3.12405

**Published:** 2020-10-16

**Authors:** Hirotoshi Kobayashi, Kenjiro Kotake, Kenichi Sugihara

**Affiliations:** ^1^ Department of Surgery Tokyo Metropolitan Hiroo Hospital Tokyo Japan; ^2^ Department of Surgery Teikyo University School of Medicine Mizonokuchi Hospital Kanagawa Japan; ^3^ Department of Surgery Sano City Hospital Tochigi Japan; ^4^ Department of Surgical Oncology Graduate School Tokyo Medical and Dental University Tokyo Japan

**Keywords:** colorectal cancer, peritoneal metastasis, R0 resection

## Abstract

**Aim:**

To investigate the usefulness of resection for synchronous peritoneal metastasis from colorectal cancer.

**Methods:**

The patients who underwent surgery for stage IV colorectal cancer at 16 hospitals between 1991 and 2007 were enrolled in this study. The overall survival rates of patients with synchronous peritoneal metastasis from colorectal cancer with and without R0 resection were compared using a propensity score‐matched analysis.

**Results:**

Among the 3965 patients with stage IV colorectal cancer, 1169 had synchronous peritoneal metastasis (28.5%). No patients received hyperthermic intraperitoneal chemotherapy (HIPEC) in this study. Among the 1169 patients, 783 had enough clinicopathologic information and went through further analysis. Out of 783 patients, 204 underwent R0 resection. A multivariate analysis revealed that severity of peritoneal metastasis according to the Japanese classification (*P* < .0001) and distant metastases (*P* < .0001) were independently associated with non‐R0 resection. In a propensity score‐matched analysis, 118 patients who underwent R0 resection were matched with 118 patients who did not undergo R0 resection. There was no significant difference in each parameter between patients with and without R0 resection. After matching, the overall survival in patients with R0 resection was better than that without R0 resection (median survival time: 28.8 months and 15.6 months, *P* < .0001).

**Conclusion:**

The overall survival of patients with R0 resection for synchronous peritoneal metastasis from colorectal cancer was better than that without R0 resection even without HIPEC.

## Graphical abstract

A propensity score‐matched analysis disclosed that the outcomes of patients with R0 resection for colorectal cancer with synchronous peritoneal metastasis were better than those without R0 resection.

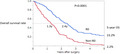

## 1 INTRODUCTION

Colorectal cancer is the second most common cause of cancer mortality in the United States and Japan.^1^, ^2^ Furthermore, the incidence of colorectal cancer is increasing rapidly in Japan.^2^, ^3^

Peritoneal metastasis is one of the poor prognostic factors in patients with colorectal cancer and is found in 5%‐10% of primary colorectal cancer cases.^4^, ^5^ The patients with synchronous peritoneal metastasis are classified into Stage IVC in the latest American Joint Committee on Cancer (AJCC) Cancer Staging Manual.^6^ There is urgent need to develop modalities to improve the prognosis of patients with colorectal cancer with synchronous peritoneal metastasis. R0 resection of both primary colorectal cancer and peritoneal metastasis has been reported to lead to better outcomes^5^. However, preoperative detection of peritoneal metastasis from colorectal cancer is difficult. The surgeons often choose operative procedures for peritoneal metastasis during surgery.

The aim of this study was to investigate the usefulness of R0 resection for colorectal cancer with synchronous peritoneal metastasis using a multi‐institutional database and a propensity score‐matched analysis.

## 2 METHODS

### 2.1 Patients

The 16 member‐hospitals of the Japanese Society for Cancer of the Colon and Rectum (JSCCR) collected the data of the 3965 patients with stage IV colorectal cancer treated by surgery between 1991 and 2007. Of these patients, 1169 had synchronous peritoneal metastasis (28.5%). Among these, 783 had detailed information, and their data were further analyzed. Neither extended peritonectomy nor hyperthermic intraperitoneal chemotherapy (HIPEC) was performed in this study. The evaluation of peritoneal metastasis was performed by surgeons during the colorectal surgery. The ethics committee of Tokyo Metropolitan Hiroo Hospital approved this study.

### 2.2 Parameters

The parameters used in this study included gender, age, location of primary tumor, histologic type, depth of tumor invasion, lymph node metastasis, hematogenous metastasis, and the extent of peritoneal metastasis. The extent of peritoneal metastasis was described according to the Japanese classification (Table 1).^7^ A previous report evaluated the objectivity of the Japanese classification as follows: P1, peritoneal metastases 20 mm or smaller confined to one area; P2, 10 or fewer peritoneal metastases disseminated in two or more areas, or peritoneal metastases confined to one area but the size is >20 mm; and P3, more than 10 peritoneal metastases disseminated in two or more areas.^8^

**TABLE 1:** Japanese classification of peritoneal metastasis from colorectal cancer

P0	No peritoneal metastasis
P1	Metastases only to adjacent peritoneum
P2	A few metastases to distant peritoneum
P3	Numerous metastases to distant peritoneum

### 2.3 Statistical analysis

Associations between patients with and without curative resection were analyzed by the *χ* ^2^ test. The actuarial survival after surgery was determined from Kaplan‐Meier curves. The log‐rank test was used to compare the overall survivals. The Cox proportional hazards model was used to determine the independent prognostic factors in patients with colorectal cancer and synchronous peritoneal metastasis.

Thereafter, pairwise 1:1 propensity score matching, including logistic regression, was used to reduce the effects of non‐random assignment of patients to curative resection. The propensity score matching method has been used to reduce potential confounding caused by unbalanced covariates.^9^ In short, by multivariate logistic regression analysis, the propensity score for curative resection was determined. Patients with and without curative resection were matched by greedy matching without replacement.

The SPSS 22 software (IBM) was used for data analysis. Median and its range or numbers of patients and ratios (%) were expressed as data. *P* value <.05 indicated statistical significance in this study.

## 3 RESULTS

### 3.1 Patient characteristics

Table 2 shows the patient characteristics for the entire cohort. The median age was 63 years in patients with and without R0 resection, respectively. Among the eight parameters, location of tumor (*P* = .0091), distant metastasis (*P* < .0001), and extent of peritoneal metastasis (*P* < .0001) had significant differences between patients with and without R0 resection for colorectal cancer with synchronous peritoneal metastasis. The smaller extent of peritoneal metastasis (*P* < .0001) and the absence of distant metastasis (*P* < .0001) were independently associated with R0 resection. In 481 patients with distant metastasis, the number of patients with liver metastasis only, both liver and other distant metastasis, and distant metastasis other than liver was 250, 132, and 99, respectively. The R0 resection rates in the three groups were 11.6%, 2.3%, and 23.2%, respectively.

**TABLE 2:** Characteristics of the entire cohort (783 patients)

	Univariate analysis			Multivariate analysis		
	Curative resection (+) (N = 204) (%)	Curative resection (‐) (N = 579) (%)	*P* value	Odds ratio	95% CI	*P* value
Age	63 (33‐91)	63 (26‐96)	.6			
Female gender	106 (52.0)	268 (46.3)	.16			
Location of primary tumor						
Colon	166 (24.4)	514 (75.6)		1		
Rectum	38 (36.9)	65 (63.1)	.0091	1.24	0.76‐2.02	.39
Histologic type						
Well or mod	159 (27.1)	428 (72.9)				
Others	45 (27.0)	151 (77.0)	.25			
T‐category						
<T3	58(29.6)	138 (70.4)				
T4	146 (24.9)	441 (75.1)	.2			
N‐category						
N0	36(29.3)	87 (70.7)				
N1a	27 (27.8)	70 (72.2)				
N1b	39 (28.9)	96 (71.1)				
N2a	36 (21.9)	128 (78.1)				
N2b	66 (25)	198 (75)	.56			
Distant metastasis						
Absent	149 (49.3)	153 (50.7)		1		
Present	55 (11.4)	426 (88.6)	<.0001	0.20	0.12‐0.31	<.0001
Peritoneal metastasis						
P1	133 (41.2)	190 (58.8)		1		
P2	59 (27.3)	157 (72.7)		0.49	0.33‐0.73	.0003
P3	12 (4.9)	232 (95.1)	<.0001	0.062	0.031‐0.11	<.0001

Abbreviations: CI, confidence interval; Well or mod, well or moderately differentiated adenocarcinoma.

### 3.2 Survival

**FIGURE 1:**
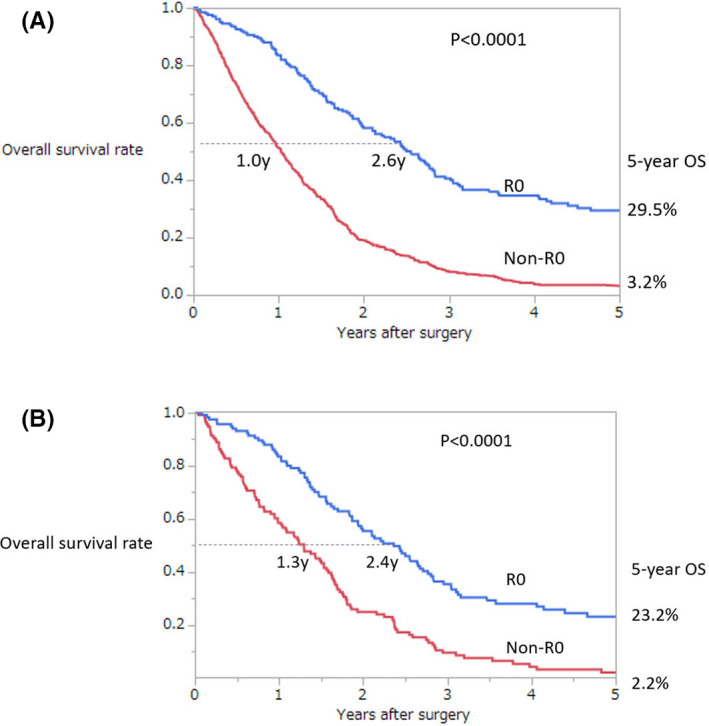
Overall survival curves of patients with and without R0 resection for synchronous peritoneal metastasis from colorectal cancer before (A) and after match (B)

The median follow‐up period of the entire cohort was 14.4 (0‐206.4) months. The median survival time (MST) of patients with and without R0 resection was 27.6 months and 10.8 months, respectively (*P* < .0001). There was a significant difference in overall survival between patients with and without R0 resection (*P* < .0001). The 5‐year overall survival rates with and without R0 resection were 29.5% and 3.2%, respectively (*P* < .0001, Figure 1A). The 5‐year survival rate of patients with P1, P2, and P3 peritoneal metastasis was 14.5%, 11.5%, and 4.0%, respectively.

### 3.3 Prognostic factors

The factors associated with prognosis by log‐rank test were histologic type (*P* = .0061), regional lymph node metastasis (*P* < .0001), distant metastasis (*P* < .0001), and peritoneal metastasis (*P* < .0001, Table 3). Among these factors, histologic type (*P* = .015), regional lymph node metastasis (*P* = .045, N1a; *P* = .0034, N1b; *P* = .014, N2a; *P* < .0001, N2b), distant metastasis (*P* < .0001), and peritoneal metastasis (*P* < .0001) were independent factors for overall survival (Table 3).

**TABLE 3:** Prognostic factors of the entire cohort (783 patients)

Characteristics	Log‐rank test		Cox proportional hazards model		
	Number of patients	*P* value	Hazard ratio	95% CI	*P* value
Age	63 (26‐96)	.31			
Gender					
Male	409				
Female	374	.43			
Location of primary tumor					
Colon	680				
Rectum	103	.91			
Histologic type					
Well or mod	587		1		
Others	196	.0061	1.26	1.05‐1.52	.015
T‐category					
<T3	196				
T4	587	.14			
N‐category					
N0	123		1		
N1a	97		1.36	1.01‐1.85	.045
N1b	135		1.52	1.15‐2.00	.0034
N2a	164		1.4	1.07‐1.82	.014
N2b	264	<.0001	1.89	1.48‐2.42	<.0001
Distant metastasis					
Absent	302		1		
Present	481	<.0001	1.94	1.65‐2.30	<.0001
Peritoneal metastasis					
P1	323		1		
P2	216		1.14	0.94‐1.38	.19
P3	244	<.0001	1.56	1.31‐1.90	<.0001

Abbreviation: Well or mod, well or moderately differentiated adenocarcinoma.

### 3.4 Propensity score matching cohort

A binomial logistic regression model was used to calculate the propensity score. Extent of lymphadenectomy (*P* < .001), tumor location (*P* = .007), and distant metastasis (*P* < .0001) were selected. The Hosmer‐Lemeshow test showed a good fit for this model (*P* = .096). The C statistic of this model was 0.88 (95% confidence interval: 0.85‐0.90). In this study, 118 patients who underwent curative resection were matched with 118 patients who did not undergo curative resection (Table 4). As for each predictive parameter, no significant difference was found between patients with and without R0 resection, which showed that these two groups were well‐matched by propensity score.

**TABLE 4:** Characteristics of the propensity score matched cohort (236 patients)

	Curative resection (+) (N = 118) (%)	Curative resection (‐) (N = 118) (%)	*P* value
Age	62 (33‐91)	61 (26‐86)	.21
Female gender	53 (44.9)	55 (46.6)	.79
Location of primary tumor			
Colon	103 (49.8)	104 (50.2)	
Rectum	15 (51.7)	14 (48.3)	.84
Histologic type			
Well or mod	91 (50.3)	90 (49.7)	
Others	27 (49.1)	28 (50.9)	.88
T‐category			
≤T3	33 (47.1)	37 (52.9)	
T4	85 (51.2)	81 (48.8)	.57
N‐category			
N0	15 (44.1)	19 (55.9)	
N1a	16 (29.3)	11 (40.7)	
N1b	24 (57.1)	18 (42.9)	
N2a	21 (42)	29 (58)	
N2b	42 (50.6)	41 (49.4)	.47
Distant metastasis other than liver			
Absent	63 (52.5)	57 (47.5)	
Present	55 (47.4)	61 (52.6)	.43
Peritoneal metastasis			
P1	63 (48.5)	67 (51.5)	
P2	43 (54.4)	36 (45.6)	
P3	12 (44.4)	15 (55.6)	.58

Abbreviation: Well or mod, well or moderately differentiated adenocarcinoma.

The MST of patients with and without R0 resection was 28.8 months and 15.6 months, respectively, in the propensity score‐matched cohort. The 5‐year overall survival rates after matching were 23.2% and 2.2%, respectively. The overall survival of patients with R0 resection was better than that of those without (*P* < .0001, Figure 1B).

## 4 DISCUSSION

Peritoneal metastasis is known as a poor prognostic factor in patients with colorectal cancer. Colorectal cancer with synchronous peritoneal metastasis is classified into stage IVC by the recent AJCC Cancer Staging Manual.^6^ Since one of the reasons for the poor prognosis in patients with peritoneal metastasis is less chemosensitivity, cytoreductive surgeries with HIPEC have been developed. Cytoreductive surgery with HIPEC benefits limited patients with peritoneal metastasis.^10^, ^11^ A previous study demonstrated that aggressive procedures, including peritonectomy with HIPEC, were beneficial for patients with a peritoneal carcinoma index (PCI) score of 10 or less.^12^ Although we also reported the usefulness of R0 resection without HIPEC for peritoneal metastasis from colorectal cancer, those were retrospective studies.^13^, ^14^ Therefore, we conducted a propensity score‐matched analysis in this study to remove biases as much as possible.

In the present study, the prognoses of the patients with R0 resection for synchronous peritoneal metastasis from colorectal cancer were better than those without in propensity score‐matched groups as well as in non‐matched groups. Before matching, there were significant differences in location of primary tumor, accompanying distant metastasis, and severity of peritoneal metastasis between R0 and non‐R0 resection groups. After matching, although no differences were found in these factors, 5‐year survival rate in R0 resection group was 20% higher than that in non‐R0 group even without HIPEC.

Currently, four randomized controlled trials have been reported regarding the efficacy of HIPEC.^11^, ^15^, ^16^, ^17^ Of these, two trials were stopped before the completion of the proposed patient recruitment.^15^, ^16^ Verwaal et al^11^ reported that cytoreductive surgery followed by HIPEC improved survival in patients with peritoneal carcinomatosis of colorectal origin despite the high mortality rate of 8%. However, in their study, up‐front chemotherapy was not used. Prodige‐7, a trial with current chemotherapy for peritoneal carcinomatosis demonstrated satisfactory outcomes with the MST of 41.7 and 41.2 months in patients with cytoreductive surgery with and without HIPEC, respectively.^17^ However, the additional efficacy of HIPEC following cytoreductive surgery was not proven. In the present study, the MST of patients with and without R0 resection was 28.8 and 15.6 months in a propensity score‐matched cohort. The difference of MST between Prodige‐7 and this study might derive from the difference of two cohorts. In the present study, the majority of patients had hematogenous metastasis as well as peritoneal metastasis. However, this study demonstrated that the curative resection led to better outcomes not only in patients with peritoneal metastasis alone but also in those with hematogenous and peritoneal metastasis.

The patients with colorectal cancer and synchronous peritoneal metastasis often have synchronous hematogenous metastasis. In this study, 61.4% of patients had hematogenous metastasis with peritoneal metastasis. R0 resection for both hematogenous and peritoneal metastases is considered difficult. The R0 resection rate for both peritoneal and hematogenous metastases was 11.4% in the entire cohort, although that for patients without hematogenous metastasis was 49.3%. Since this was a multi‐institutional study, the treatment strategy depended on each institution. However, the Japanese guidelines for the treatment of colorectal cancer recommended the R0 resection for colorectal cancer and synchronous metastasis if the surgical stress is not too much. The factors independently associated with R0 resection were the Japanese classification of peritoneal metastasis (P1 and P2) and the absence of hematogenous metastasis. These results disclosed that the Japanese classification is easy‐to‐use as well as useful in selecting patients for R0 resection during the surgery. The patients with P1 or P2 peritoneal metastasis should undergo R0 resection if distant metastasis is not found in preoperative screening.

The MST of patients with R0 resection was 27.6 months in the present study. The MST of patients with cytoreductive surgery with HIPEC was reported to be 12 months to 62.7 months in previous studies.^4^, ^24^ The median survival of 26 previous studies was 20 months. Although some of these studies reported good outcomes, metachronous peritoneal metastasis was included as well as synchronous metastasis. Therefore, it is hard to compare these outcomes. The present study disclosed the low R0 resection rate in patients with both peritoneal and hematogenous metastases from colorectal cancer. The treatment for these patients needs improvement. The current multi‐agent chemotherapy with molecular‐targeted drugs has improved the outcomes of patients with advanced colorectal cancer.^25^, ^26^ However, the most appropriate regimen for peritoneal metastasis from colorectal cancer is still unknown. It should be clarified in the future.

At present, R0 resection is the best way to cure patients with colorectal cancer with synchronous peritoneal metastasis. To improve the outcomes of those patients, it is important to increase the number of patients with R0 resection. We previously reported that R0 resection of peritoneal metastasis from colorectal cancer led to better survival in patients with synchronous peritoneal metastasis from colorectal cancer even without HIPEC.^5^ Additionally, a randomized controlled study demonstrated that cytoreductive surgery plus HIPEC led to better outcomes than palliative surgery.^11^ Elias et al demonstrated an excellent outcome with a 5‐year survival rate of 27% after the cytoreductive surgery with HIPEC.^10^ Cytoreductive surgery with HIPEC for patients with colorectal cancer with peritoneal metastasis is performed in many peritoneal centers as gold standard.

The present study clarified that histologic type, regional lymph node metastasis, hematogenous metastasis, and the extent of peritoneal metastasis were independent prognostic factors. Therefore, even if patients with peritoneal metastasis undergo R0 resection, those with regional lymph node metastasis, hematogenous metastasis, poorly differentiated adenocarcinoma, and P3 peritoneal metastasis might need powerful adjuvant chemotherapy. The development of more effective drugs and regimens are desired to improve the prognosis in patients with peritoneal metastasis from colorectal cancer.

There were some potential limitations in this study. First, although the reproducibility of the Japanese classification of peritoneal metastasis from colorectal cancer was validated in a previous study,^8^ it seems prone to interobserver differences. Second, since the present study was a retrospective one, there might be biases. In fact, the data of patients without surgery were not included in this study. Third, cytoreductive surgery with HIPEC was not performed in the present study, because these were not standard procedures in Japan. This might result in the low R0 resection rate of P3 patients. Fourth, the number of patients who could not be matched with a propensity score was large in the present study. The reason for this could be a small number of P3 patients with R0 resection. Fifth, the study period is relatively old. The role of R0 resection in the era of modern chemotherapy with molecular target agents should be examined in the future.

In conclusion, curative resection for synchronous peritoneal metastasis from colorectal cancer could lead to better outcomes even without HIPEC. The absence of hematogenous metastasis and the P1‐P2 peritoneal metastasis were most favorable factors benefiting from synchronous resection of peritoneal metastasis. These data suggest that we need a better study comparing HIPEC to modern systemic chemotherapy following complete resection of peritoneal disease.

## DISCLOSURES

## Conflict Of Interest

There is no conflict of interest in this study.

Funding: This study was supported by the Japanese Society for Cancer of the Colon and Rectum.

Author contribution: H. Kobayashi study design; acquisition, analysis, interpretation of data, drafting the manuscript. K. Kotake and K. Sugihara: interpretation of data, critical revision. All authors approved the final version.
